# Medicine*Read before the Organon and Materia Medica Club of the Bay Cities of California, October 5th, 1894.

**Published:** 1894-11

**Authors:** J. M. Selfridge

**Affiliations:** Oakland, Cal.


### MEDICINE.*

J. M. Selfridge, Oakland, Cal.

It would be interesting, did time permit, to commence, if we could, at the initial point of medical practice and trace the various improvements that have occurred from the time that medicine was first administered down to our own time. This, however, would be both prolix and difficult, for the advent of practical medicine is so obscured by the dim light of the distant past and so deeply covered by the dust of ages that it would be almost, if not quite, impossible to go back and unfold the leaves of Time sufficiently to throw very much light on the *origin* of our subject. The necessity for medicine presupposes the presence of disease, and, without doubt, disease is the result of violated physiological law. We are told that man was made in the image of his Creator, and, therefore, mentally and physically perfect. However true this may be, the history of the race shows that he was created with capabilities that soon got him into trouble, and his offspring, in a very few years, needed doctors, and they have had need of them more or less ever since, and, I have no doubt, will continue to need them, not only down *to* but clear *through* that blissful time when the nature of men and animals, it is said, will be so changed that the lion and the lamb will become good friends, and the lion will be so modified in his tastes that he will prefer to eat straw rather than lamb-chops.

Although the necessity for doctors was, doubtless, coeval with the human race, yet the earliest records we have are so mixed with astrological nonsense and Druidical incantations that we are compelled to set them aside, for they are of little value. From the best lights we have, we learn with strong probability that the Egyptians were the first to give to the world a knowledge of medicine. We now know that the Egyptians were

---

*Read before the Organon and Materia Medica Club of the Bay Cities of California, October 5th, 1894.

practicing medicine and writing treatises on medical subjects more than twenty centuries before the Christian era. Eleven hundred years before the winged messengers of the *Most High* announced, with glad hosannas, the birth of a World's Redeemer to the shepherds on the plains of Bethlehem, the first medical college was erected in Egypt, and dedicated to the education of men in the art of healing. Too little is known, however, of their methods to enable us to form any opinion of the value of their teaching or the success of their practice.

From Egypt a knowledge of medicine was wafted to Æsculapius and Hippocrates in Greece; thence to Celsus and Galen in Rome, the latter of whom established a theory and practice of medicine that is well formulated in the Latin words "*Contraria contrariis curantur.*" His system partook so strongly of his own energetic nature that it has held more or less sway down through the ages, and is not even now entirely dispelled by the light of the nineteenth century. Harvey, Sydenham, Bonet, Morgagni, Boerhave, Haller, and a host of others have borne testimony to the strength and genius of Galen, and, like him, their course has been written in the blood of their victims.

Medicine has never kept pace with the advancement in the arts and sciences and other branches of human knowledge. If proof of this were necessary, a retrospective glance at the perfection of architectural beauty in ancient Greece and the attainments in letters of both Greece and Rome would be ample testimony. But we need not go back to those ages of civilization long since dead, for it is a matter of history that in the eighteenth century *medicine* was in its *darkest* hour. If we examine the medical literature of that period, we find from twenty to thirty remedies compounded in the same prescription. Verily, it might, with propriety, be called the age of drugging. Not satisfied with the profuse administration of drugs internally, all sorts of external applications were used, until it would seem to be the height of their ambition to devise something never before heard of, and in some instances the most abominable and repulsive applications were used which the human intellect could invent. Think, for example, of a delicate young woman, suffering with peritonitis, being subjected to the application of the warm entrails of a sheep, disembowled in her own bed-room for that purpose. Think of another young woman, suffering with a contraction of the hamstring muscles, being subjected for over forty days to the application of a *cataplasma de sterecore humano.* Disgusting though these examples are, they are but feeble expressions of the condition of medicine, when, in the latter part of the eighteenth century, the light of scientific truth first flashed like a meteor across the horizon of the therapeutic world.

It was in 1790 that Hahnemann took Cinchona and watched its effects. To his analytical mind it was more than the simple effect of a medicine. Like the falling of an apple to the eye of a Newton, it opened to the mind of Hahnemann a new field of thought—a new laboratory was constructed, in which he labored until he gave to the world the law of *similars,* which is the *law of God.* The observations of Hahnemann have since been verified by hundreds, yes, thousands, of able, honest, industrious men and women; and yet, in the light of this nineteenth century, with the experiences of past decades spread out before them, with an abundance of opportunities to verify or disprove, if they can, the statements of Hahnemann, there are hundreds of physicians, who, without examination, pronounce Homœopathy a delusion and snare. If these villifiers were all in the ranks of the allopathic school, we might pass them with a smile at their ignorance. But, when we find those who are counted as homœopaths call in question, both by example and by precept, the truths as announced by Hahnemann, as we have heard done by those who are professed teachers of Homœopathy, is it not time for honest homœopaths to call a halt and inquire of these *croakers* as to which "pathy" they belong, whether eclectic, allopathic, or a compound of the three?

The truth or falsity of Homœopathy is susceptible of demonstration; hence it seems to me eminently proper that those who assume the name for the money there is in it should in some way be made to feel that common honesty required them to examine carefully and conscientiously the principles as laid down in Hahnemann's *Chronic Diseases* and the *Organon of Homœopathy*, and if they find them a delusion and a snare, which their own practice and teachings would lead us to believe they think, let them so announce themselves, so that *we* may know—so that the *world* may know that they are not *practical hypocrites.* Homœopathy has suffered many wrongs, but none of its wounds are so deep as those inflicted by professed friends. In other words, the physician who claims to be a homœopath, and still resorts to allopathic methods in the treatment of disease, is *not* what he professes to be. "He is wounding Homœopathy in the house of its friends." If he knows no better, he is to be pitied but *not* excused, for he *ought* to know better—he *can* know better if he will but apply himself to study. Homœopathic physicians ought to be so proud of the title, and so satisfied with their own *armamentarium*, that they would not care to seek after the gods of the Philistines, for it is well known that they are better equipped and better able to cure disease with remedies which are not only safer and surer, but entirely different from those of the old school.

Not many weeks ago I was informed by an intelligent lady from the East that she had understood there was not a pure homœopathic physician west of the Rocky Mountains. Need it be asked why this is so? Clearly, the physicians who violate the principles and practice of pure Homœopathy so generally are responsible for giving so *humiliating* an impression to the public. Is it any wonder that the allopaths speak disrespectfully of Homœopathy when they find its representatives giving crude Calomel, etc., in the form of tablets? To be a good homœopathic physician does not necessarily require him to be a high-potency man. On the contrary, his practice may be strictly in accordance with the law of similars, even though he administer the mother tincture. And, *vice versa*, a physician may use only the high or even the highest potencies, and not be a practitioner of Homœopathy, for the simple reason that he does not select his remedy in accordance with the law of similars.

Again, a physician, to be a progressive homœopath, should *never* alternate his remedies. The reason is very apparent—he never can know which remedy cures, and, therefore, *never learns* anything. But he who prescribes in accordance with the law of similars, and gives only the single remedy, will, sooner or later, go higher and higher in the scale of potencies. This, at least, is my *personal* experience. At one time I was the *crudest* of the *crude*, and alternated remedies without rhyme or reason, but gradually, by a process of evolution, it may be, I am now a firm believer in the efficacy of the high and even of the *highest* potencies. There is probably not a homœopathic physician living who will deny that *low* potencies do sometimes make *cures*, while there are hundreds, and, perhaps, thousands, who conscientiously believe that there is *no* medicine in high potencies, and, therefore, no therapeutic power. Olmstead, in his work on *Natural Philosophy*, says a particle of matter cannot be so finely pulverized that it may not be again divided. High-potency men go beyond this. They do not claim that there is *any material* substance in the highest potencies—nothing, in fact, but medicinal force *dynamitized.* This idea is ridiculed by the materialists; in fact, I am not aware that they admit there is such a thing in existence as dynamic force. There is only one situation in which I can place these objectors, and that is in company with those ancient astronomers who believed that the earth was flat and rested upon the back of a huge turtle.

My dear objector, did you ever look through a telescope at Saturn and his beautiful rings, or Jupiter with his five moons? Did you ever stop to think of how many thousands of billions of tons of rock and other material are contained in one of these huge planets? Upon what do they rest? If upon nothing, what holds them suspended in mid-air? The force of gravity, say you? Be it so. But what *is* the force of gravity? You do not know? Neither do I. But, from what I have seen and know, it is my firm belief that it is one of the dynamic laws of the Creator of this great universe. Dynamic force pervades *all* things. In other words, dynamic force, in one way or another, *creates*, *upholds*, and *controls*, not only our own bodies, but every *particle* of matter, whether animate or inanimate, in this broad domain of infinite distances. This force, in the planetary system, reaches millions of miles from one planet to another—influences and, to a certain extent, controls their movements. This being true, is there anything unreasonable in the doctrine that the force which pervades all medicines, as well as other substances, can be detached from its material mother and *attached* to another material? As, for example, alcohol, water, or sugar of milk.

To my mind there is nothing unreasonable in what we know to be a fact, and that it is a fact, there are hundreds who can bear testimony. The education of any person who doubts it has been sadly neglected, or prejudice has been allowed to blind the understanding. By patient investigation, and the conscientious application of principles, all honest inquirers need not be long in doubt. There is probably nothing in the entire range of the homœopathic system that has met with such bitter opposition as the use of high potencies and what is claimed for them. If ever mud was thrown at any object, if anything was ever ridiculed, if anything was ever placed among the most unscientific nonsense—the absurdest of all absurdities—the high-potency idea is that thing. And among all the opponents, ridiculers, mud-throwers, and dogmatic disbelievers in them, I, myself, have been the chief.

But, Mr. President, as the light of divine truth caused the scales to fall from the eyes of St. Paul of old, so, also, has the light of pure Homœopathy penetrated my hitherto obdurate mind and caused the scales of dogmatic prejudice to fall from the eyes of my darkened understanding. It has given me the courage to investigate, to experiment, to prove all things and hold fast to that which is good. From massive doses of Calomel, Jalap, Rhubarb, and Quinine, I have ascended, step by step; from the mother tincture to the 3d, the 6th, the 30th, the 200th, the 1000th, the CM, the MM, and the DMM. I have proved them all and have gotten good results from the low as well as the high. But candor compels me to bear this testimony. For promptness of action, for well-defined, clean-cut lines and brilliant results, the well-selected remedy in the *highest* potency is the most satisfactory. A few examples of their action, and I will close this paper.

A gentleman, aged sixty-four, called at my house on Sunday, July 22d, 1894, when the following history was obtained :

When young his hair was red (it is now white); complexion florid ; medium height ; stout ; thin, white skin ; bleeds easily ; inherits cancer (his mother and sister died of it). Has a hard seed-wart on one of his fingers, and a dry crust firmly attached on the bridge of his nose, which he fears is a cancer ; was scaly for a year, during which time he has been applying zinc ointment, which drives it partly away, but it soon returns. His head gets dizzy if he leans back or stoops forward. Is disposed to fall forward. It comes on suddenly ; feels it when he coughs or lies down, and is worse if he rises suddenly from the recumbent posture. Greasy, highly-seasoned food and salads disagree. He uses tobacco ; is fond of coffee and drinks some whisky. Has itching hæmorrhoids ; itch worse at night. When he undresses his skin, in general, itches, especially under the arms, where he breaks out like hives. Had hives badly years ago. His hands and arms go to sleep when he lies on them at night. His feet cramp at times. Is easily worried about his business, and has had erysipelas twice. Here was evidence of a scycotic miasm. Whether personally contracted or inherited, I was unable to ascertain. The case being somewhat complicated, I concluded to work it out with Bœnninghausen's Pocket Book and Yingling's check-list. When the checks were counted, Sulphur stood 60, Rhus, 52 ; Sepia, 50 ; Calc-c., 49, and Thuja, 36. Although not a very close fit constitutionally, still, as Sulphur headed the list, on July 24th I gave him one dose of Sulphur $^{cm}$, with blanks to follow. These were renewed twice, but not another dose of medicine was given. As he is a busy man, his wife reported on September 8th that the supposed cancer had disappeared, and the wart also, and in other respects he is better than for a long time.

*Another case :* A lad fourteen years old came to my office complaining of his left foot, and especially of his toes. They looked as if scalded, and especially between them. In addition to this, to say they smelt badly does not express it—they positively stunk. These, with his other symptoms, corresponded so closely to Sulphur that, as an experiment, I gave him, while in the office, one powder, on his tongue, of the CM potency, with blanks to follow, and requested him to report in a week, which he did, saying his foot had stopped stinking and was almost well. Blanks were continued, with instructions to report if not entirely cured. He is now all right.

Pulsatilla, as you know, is said to act best in "persons of indecisive, slow, phlegmatic temperament; sandy hair, blue eyes, pale face; easily moved to laughter or tears; affectionate, mild, gentle, timid disposition." As an illustration of the opposite condition, I will report the following case:

Mrs. H., dark, coarse hair, dark-brown eyes, coarse skin with black pores, large frame, strong features, and resolute appearance, came into my office over two months ago and begged me to give her something to cure a terrible sick headache to which she had been a martyr almost every week from her early girlhood. Without going into details I will state the character of the pain was this: The pain recurs in paroxysms, increases to an intense point of severity, then decreases to a complete cessation. She always enjoys herself best in the open air. As Pulsatilla seemed to be indicated, and wishing to experiment with high potencies, I gave one powder, on the tongue, of the CM, with blanks to follow. A week after this her husband came in, saying, "That medicine acted like a charm on my wife, as the pain left her before she got out of the building." He wanted some of the same, as she had a mild return. One dose of the CM potency was given him, with blanks to follow. It relieved the pain promptly, and there has been no recurrence of it for two months, although she has been to the theatre, which heretofore had *always* provoked an attack of headache.

About two weeks ago I was summoned to the bedside of a patient who had suffered all night with cutting pains, recurring in paroxysms, which she referred to the region of the sigmoid flexure of the descending colon. As she had some knowledge of the action of medicines, she had taken Colocynth all night, but without relief. She could not see why she was not better, as Colocynth had these paroxysmal cutting pains among its symptoms. But my patient had looked at but one side of the picture. She had other symptoms, prominent among which was this: she had frequent desire to stool, but when she went to the closet she could accomplish nothing. Nux-vomica[cc] cured her very promptly. The allopaths, or their imitators, would doubtless have given one of the coal-tar products or a hypodermic injection of Morphine, thus disordering the system and preparing the way for more medicine.

Not long ago I was called to a lad, aged fourteen, who was taken in the night with a chill, followed by fever, soreness in the left chest, cough, with rust-colored sputa and restlessness. He could lie in one position but a short time, when he had to change to get relief. An examination discovered moist râles over the lower lobe of the left lung and in the centre of the lobe, prolonged expiratory bronchial murmur, followed with a peculiar re-active puff which is so characteristic of commencing hepatization. The case was, of course, pneumonia. The peculiar restlessness, taken in connection with his other symptoms, led me to the choice of the remedy. I gave Rhus-tox.[cc] in water, a teaspoonful every two hours—not because Rhus is said to be the *epidemic remedy*, but because it was indicated by the symptoms. On the evening of the third day his pulse was 126, respiration 36, and the temperature 104.2°, with some delirium. He appeared to be growing worse, but I had faith in the choice of the remedy and did not change it. The result proved that my confidence was not misplaced. Before morning the delirium ceased, the fever began to subside, and my patient fell asleep. When I made my visit in the morning, he was in every way better. His pulse was 80, and his temperature sub-normal, being 96°. The hepatization was rapidly clearing and the rust-colored sputa had changed to muco-purulent matter. The remedy was continued at longer intervals, and on the seventh day I discharged the patient cured. There is one point to which I wish to call your attention *especially*. There were no external applications of any kind or description. A flannel shirt was the only addition to his ordinary night-dress. This, then, was a clean-cut homœopathic cure in a shorter time by weeks than anything of which the best allopathic practice can boast, and without any risk to the patient.

The following case has interested me greatly for the reason, among other things, that it crosses the track of the gynæcological surgeon, and shows what may be done in desperate cases without the knife. This, to my mind, was one of those constitutional cases upon which to have operated would have led to fresh disaster.

About six months ago Mrs. S., aged about thirty, married eight years, but no children; tall, with brown hair, gray eyes, and fair skin that freckles, was sent to me by a mutual friend. Two years before she had been under the care of one of the foremost allopathic gynæcologists of San Francisco, who was treating her for leucorrhœa and "disease of the right fallopian tube," when he discovered "a tumor about as large as a pea" in the right broad ligament, and advised an operation. When she came to me it was about the size and shape of a small pear. That is to say, it was about two and one-half inches in its long diameter by one and a half inches at its broadest point, with no adhesions. There was also a tumor the size of a small marble on the front surface of the womb about half an inch above the anterior fornix. The womb was about the normal size, possibly enlarged a little, but harder than in health. The mucous membrane of the external cervix was denuded, was red, and the slightest touch caused the granulations to bleed. The endometrium was in a state of chronic inflammation, which caused a very profuse, acrid, tenacious leucorrhœa, having an unpleasant odor, to pour from the interior of the womb. The womb and appendages were so tender that she could not bear the embraces of her husband without great suffering. The menses were somewhat irregular, but not profuse. When she was in her seventeenth year she had several hæmorrhages from her lungs, and her mother and sister have since died of consumption. An examination discovered the presence of latent tubercles at the apex of the right lung. To have operated on a case with such decided tuberculous cachexia, without first placing the system in as favorable a condition as possible, would, to my mind, have been a surgical murder. Consequently, after some study, I gave her Tuberculinum$^{dmm}$, one dose on the tongue, with blanks to follow. She has taken four such doses at intervals of about a month. I have found, what I presume has been the experience of other homœopathists, that patients afflicted with uterine troubles always expect some kind of local treatment. In fact, it is almost impossible to hold them unless something in that line is done. Hence, with this in view, and with the hope of assisting in the depletion of the engorged tissues, I applied tampons of absorbent cotton, moistened with glycerine, to which was added enough Carbolic acid to prevent decomposition. These were placed in position twice a week, except during the menstrual effort, and douches of hot water ordered daily in the interim. I am fully aware that the result obtained will be called in question because these appliances have been used. In other words, due credit will not be given the remedy administered, and perhaps with some show of justice, for, in Hering's *Guiding Symptoms* we find among the cured symptoms of Carbolic acid : " Induration and ulceration of the cervix uteri, copious discharge of fetid, greenish, acrid matter from the vagina." Carbolic acid, however, does not cover the case, and, therefore, would require some stretch of the imagination to be considered the simillimum in this case. Of one thing I am very sure: there is probably no gynæcologist living who would have considered the use of tampons sufficient in this and similar cases, without first having curetted the womb, and then performed cœliotomy to get rid of the tumor. Such treatment would, in my opinion, have developed the latent tubercles now in her lungs.

Without further delay I give you the result. I have been unable to detect any growth of the tumor since the eighth week of the treatment. The catarrho-septic condition of the womb is entirely removed. The cervical mucous membrane is completely healed, and the parts, instead of looking red and granulated, are normal in color and healthful in appearance. The leucorrhœa and tenderness of the womb and ovaries have entirely disappeared, and she is now able to receive the embraces of her husband without the slightest discomfort. For the last two months she "has been feeling better than at any time since her marriage," now over eight years. All this encourages us to feel that the case is completely cured. Of course, the tumor is still there, but if it never enlarges and the system continues to tolerate it, is she not cured? Time alone will tell.

The cure of diseases with high potencies is a problem that is difficult for the human mind to grasp. Men have been so accustomed to dwell upon things material that to think of anything other than material substances being able to bring strength out of weakness and health out of disease is to them an unreasonable proposition. In considering this question, however, we must not lose sight of the fact that the most powerful forces in Nature are, so far as we are able to demonstrate, without material form. Just how or why these forces act as they do, or *how* one force supplants another in the cure of disease, is difficult to comprehend, but no more so than how the force we call gravity holds planets, which are millions of miles from each other, in their orbits, while at the same time it causes them to move with the utmost precision from the beginning to the end of time. I confess it is a difficult thing for a person who has spent years in the study of material things to leave his lifelong idols and soar away from the finite to the infinite.

With truth, it has been said that the question of high potencies has driven many a person away from Homœopathy. But, if we can cure with high potencies what cannot be cured with the low, are we to cease curing our patients because there are those who cannot comprehend the *modus operandi?* They tell us these potencies are not what is claimed for them. I have heard it stated with apparent candor that the CM of Swan or Fincke were no higher than the ordinary sixth or ninth potency. It has also been said that there was nothing in them; that they are nothing more than bottle-washings. If this be so, how is it that harm not infrequently results if potencies above the 50 M are repeated oftener than once in two, four, or six weeks, when lower potencies can be repeated hourly or weekly, as the case may require, with impunity? Results are what we are after. Hence, it matters not how these potencies are made, whether by the fluxion process of Fincke or the emptying methods of others. The marvelous cures which are daily being made with them is sufficient answer to all objectors.

